# Potential exposure to endocrine disrupting chemicals and selected adverse pregnancy outcomes: a follow-up study of pregnant women referred for occupational counselling

**DOI:** 10.1186/s12995-017-0152-y

**Published:** 2017-03-09

**Authors:** Jessica Bengtsson, Pernille Søgaard Thygesen, Linda Kaerlev, Lisbeth E. Knudsen, Jens Peter Bonde

**Affiliations:** 1 Department of Occupational and Environmental Medicine, Frederiksberg and Bispebjerg Hospitals, Bispebjerg Bakke 23, Copenhagen, NV DK-2400 Denmark; 2 Research Unit of Clinical Epidemiology, Institute of Clinical Research, University of Southern Denmark, Odense, Denmark; 3 Center for Clinical Epidemiology, Odense University Hospital, Odense, Denmark; 4 Department of Public Health, Section of Environmental Health, University of Copenhagen, Copenhagen, Denmark

**Keywords:** Endocrine disrupting chemicals, Birth weight, Gestational age, Job exposure matrix, Occupational health clinic, Work health, Prospective study, Epidemiology, Public health

## Abstract

**Background:**

Experimental evidence indicates that fetal exposure to xenobiotics with the potential to interfere with the endogenous steroid hormone regulation of fetal development may reduce birth weight. However, epidemiological studies are limited. The aim of the study was to investigate whether potential occupational exposure to endocrine disrupting chemicals (EDC) of the mother during pregnancy is associated with preterm birth and low birth weight.

**Methods:**

Pregnant women referred to an Occupational Health Clinic (OHC) in two Danish regions (Copenhagen or Aarhus) between 1984 and 2010, suspected of being exposed to occupational reproductive hazards were included in the study. A job exposure matrix enabled estimation of potential occupational exposure to EDC on the basis of job title. Births by women potentially exposed to EDC (*n =* 582) were compared to births by women referred to an OHC on the suspicion of other exposures than EDC (*n =* 620), and to a sample of births by all occupationally active women in the same geographical regions (*n =* 346,544), including 1,077 births of the referred women’s non-referred pregnancies.

**Results:**

No indications of reduced birth weight or increased risk of preterm birth were found among women potentially exposed to EDC. Women potentially exposed to EDC had children with a higher birth weight compared to the sample of occupationally active women but not compared to other women referred to an OHC.

**Conclusions:**

Potential maternal exposure to EDC at Danish workplaces is not related to low birth weight or preterm birth among women referred to occupational counselling. Occupational exposures might be too weak on the average to cause these adverse effects or counselling at the OHCs is effective in preventing them.

## Background

Endocrine disrupting chemicals (EDC) are exogenous substances that cause adverse health effects through interference with the endocrine system [1, 2]. EDC may affect the endocrine system by mimicking or blocking the action of an endogenous hormone or bind to transport proteins. Furthermore, EDC may interfere with the normal endocrine system by changing the normal hormone level through stimulation or inhibition of the production of hormones [1, 2]. Several epidemiological studies suggest that a range of EDC may actually cause preterm birth and/or low birth weight [3, 4]. Many of these studies are concerned with specific substances, such as phthalates [5], some pesticides [6, 7] and poly-chlorinated biphenyls (PCB) [8]. For example, exposure to PCB is believed, with increasing evidence, to inhibit fetal growth [8]. Due to methodological restrictions, the complexity of these chemical substances, and due to preventive measures, the effects are often limited and the results inconsistent [9, 10]. Therefore, it is difficult to determine whether associations between EDC and preterm birth and/or low birth weight are causal [11, 12].

Denmark has had regulation on worker protection since 1873. According to the Danish Working Environment Act, the employer is responsible for ensuring a safe and sound working environment (Executive Order no. 1072 of 7 Sept. 2010). If a potentially harmful reproductive risk is present at the workplace, including exposure to EDC, pregnant women may be referred to an Occupational Health Clinic (OHC) by their general practitioner or midwife for reproductive risk assessment. This usually happens between 8 and 12 weeks of gestation. The clinics perform a risk assessment based upon available evidence in order to assess if the pregnant woman may continue in her job or whether there is a case for intervention in terms of actions to reduce or eliminate hazardous exposures, restructuring of job tasks, reassignment of job functions or – as the ultimate and least desirable solution - maternity leave due to reproductive risks that could not be resolved otherwise [13, 14]. Referred pregnant women are relocated or granted sick leave until the potential risk has been determined. However, keeping the pregnant women in their job, if possible, may be beneficial for the women in order to maintain income and attachment to the labor market.

Assuming that pregnant women referred to an OHC have a more hazardous occupational setting compared to pregnant women in general, since they are referred on the basis of a concrete suspicion of a harmful risk factor, these women constitute a relevant population to investigate. The national and complete coverage of the Danish Birth Register enable comparison of birth weight and gestational age of births by women referred to these clinics to all births in the same geographical regions. The aim of the present study was to examine if potential occupational exposure to EDC during pregnancy is related to preterm birth and/or low birth weight.

## Methods

### Study population

The study population consists of pregnant women referred to an OHC for occupational counselling in two Danish regions (Copenhagen and Aarhus) in the 26 year period from 1984 to 2010. A sample of all occupationally active women giving birth in the same time period and the same geographical regions, constitute an external reference group. We made restrictions as to those who were working at the onset of their pregnancy, mother’s age at delivery 16–45 years, birth weight 1,000–7,000 g and gestational age 154–310 days. Furthermore, one birth was chosen by random sampling among women with two or more deliveries in each of the groups, which left 1,202 women referred to an OHC (261 from Copenhagen, 941 from Aarhus), and 346,544 in the external reference group including 1,077 births of the referred women’s non-referred pregnancies.

Among women receiving occupational counselling, 582 were referred on the basis of suspected exposure to EDC (defined below) and 620 were referred due to other exposures. These 620 pregnancies constitute an internal reference group.

Information about the study population obtained from the OHCs was linked to data from *Statistics Denmark* [15] and *the Medical Birth Registry* [16]. These links enabled comparison of gestational age and birth weight between the women referred to an OHC and the external reference group.

### Exposure assessment

Exposure status of referred women was determined by using a job-exposure matrix (JEM) developed by van Tongeren et al. [1]. The JEM includes 348 job titles according to the Categories of Occupation from 1980 (CO80). When developing the JEM, three occupational hygienists classified the likelihood of exposure to seven different substance groups with suspected endocrine disrupting properties (pesticides, polychlorinated organic compounds, phthalates, alkyl-phenolic compounds, bi-phenolic compounds, heavy metals and other substances) for all job titles independently. The authors defined the likelihood of exposure as *unlikely*, *possible* or *probable* [1].

Women referred to an OHC have been assigned a Danish occupational code. The code for each woman was translated into one of the job titles in the JEM. All women possibly or probably exposed to EDC in their occupation, according to the JEM, were combined into one group labelled potentially exposed*.* The seven substance categories in the JEM were combined into one overall group of EDC.

Every person with permanent residence in Denmark is given a unique 10-digit Personal Identification Number (PIN) [17]. In the present study, the PIN enabled linkage between data from the OHCs with data from Statistics Denmark, which provided information on the mothers’ age, year of births, PIN of their children, country of origin, and socioeconomic status (SES) [15]. Linkage to The Medical Birth Registry enabled access to information on birth weight, gestational age, previous pregnancies and smoking status during pregnancy [16].

### Outcome and confounder assessment

We examined the following outcomes: gestational age and birth weight, birth weight at week 40, preterm birth (<37 weeks) and low birth weight (<2500 g). The limits of preterm birth and low birth weight were set according to the WHO definition [18].

To classify the mothers into four groups of SES, DISCO-88 codes were used [19]. DISCO-88 is the Danish version of the International Standard Classification of Occupation (ISCO) and is divided into 10 main categories based on level of skills. *Group 1* (D1-2) refers to top leaders, managers and employees with skills at the highest level. Employees with skills at medium level are classified into *group 2* (D3)*. Group 3* (D4-8) contains employees with skills at basic level, and *group 4* (D9-0) contains employees with unspecified working skills. Since the DISCO-88 codes were first registered in our data from 1991, only mothers giving birth hereafter are included in this variable.

### Strategy of statistical analysis

We performed linear regression analyses of the effects of potential exposure to EDC (yes/no) on gestational age (days) and birth weight (grams) respectively while adjusting for a fixed set of potential confounding variables defined a priori: maternal age (*16*–*24*, *25*–*34* and *35*–*45* years), year of birth (*1984*–*1989*, *1990*–*1994*, *1995*–*1999*, *2000*–*2004* and *2005*–*2010)*, parity (*first*, *second* and *third or more)*, SES (*group 1*, *group 2*, *group 3*, *group 4* and *unknown)*, ethnicity (*Danish/non-Danish*) and smoking at any time during pregnancy (yes/no).

In addition to linear regression of continuous outcomes we computed odds ratios (OR) for preterm birth and low birth weight according to exposure status by logistic regression analyses. We adjusted for maternal age, year of birth, parity, SES, ethnicity and smoking habits during pregnancy.

All calculations were made by the statistical program SAS version 9.3 (SAS Institute Inc, Cary, NC, USA).

## Results

Table 1 provides the descriptive characteristics of the study population including the women potentially exposed to EDC, the internal reference group of women referred to an OHC due to exposures other than EDC, and the women in the external reference group of gainfully employed persons. The three groups are similar with respect to characteristics except that mothers referred to an OHC are younger than the external references, and tend to belong to a lower SES group.

**Table 1:** Characteristics of women with potential occupational exposure to endocrine disrupting chemicals (EDC), women unexposed to EDC (internal references) and all occupationally active women in Copenhagen and Aarhus in the time period from 1984 to 2010 (external references)

	Exposed to EDC (*n =* 582)		Internal references (*n =* 620)		External references (*n =* 346,544)	
	n	%	n	%	n	%
Maternal age						
16–24 years	106	18.2	115	18.6	44,414	12.8
25–34 years	422	72.5	417	67.3	243,236	70.2
35–45 years	54	9.3	88	14.2	58,894	17.0
Year of birth						
1984–1989	90	15.5	65	10.5	76,423	22.1
1990–1994	92	15.8	57	9.2	62,752	18.1
1995–1999	138	23.7	120	19.4	60,723	17.5
2000–2004	141	24.2	193	31.1	64,324	18.6
2005–2010	121	20.8	185	29.8	82,322	23.8
Parity						
First birth	342	59.3	329	53.8	175,945	51.0
Second birth	173	30.0	206	33.7	122,148	35.4
Third or more births	62	10.8	77	12.6	46,926	13.6
Missing	1,538					
Socioeconomic status^a^						
Group 1	31	5.3	62	10.1	49,973	14.4
Group 2	87	15.0	92	14.9	64,780	18.7
Group 3	220	37.8	274	44.5	98,666	28.5
Group 4	97	16.7	47	7.6	14,162	4.1
Unknown	147	25.3	141	22.9	118,657	34.3
Missing	310					
Ethnicity						
Danish	501	86.2	556	90.3	313,238	91.1
Foreign	80	13.8	60	9.7	30,741	8.9
Missing	2,570					
Smoking habits during pregnancy^b^						
No	364	77.9	410	76.2	206,286	80.1
Yes	103	22.1	128	23.8	51,242	19.9
Missing	89,213					

^a^ = Does only include information from 1991–2010

^b^ = Does only include information from 1996–2010

Gestational age was approximately equal across the three groups (Table 2) and linear regression analysis with adjustment for the potential confounders showed no significant effect of potential exposure to EDC on gestational age (data not shown).

**Table 2:** Crude gestational age (days) with 95% confidence intervals (CI) among referred women potentially exposed to endocrine disrupting chemicals (EDC), and among the internal and the external reference group

	n	Median	Mean	95% CI	Mean difference (95% CI)	
					Exposed versus internal reference	Exposed versus external reference
Exposed to EDC	582	280	277.5	276.4 – 278.7	−1.21 (−2.82 – 0.39)	−0.27 (−1.4 – 0.90)
Internal references	620	280	278.7	277.7 – 279.9	.	.
External references	346,544	280	277.8	277.8 – 277.8	.	.

The mean birth weight of the children born to women in the external reference group was marginally lower than the mean birth weight of the births given by women referred to an OHC, while the mean birth weight in the 40^th^ week of pregnancy was marginally lower in the group potentially exposed to EDC compared to the external references. However, the confidence intervals overlap, indicating no significant difference in mean birth weight (Table 3).

**Table 3:** Crude birth weight and birth weight at week 40 (grams) with 95% confidence intervals (CI) among referred women potentially exposed to endocrine disrupting chemicals (EDC), and among the internal and the external reference group

		n	Median	Mean	95% CI	Mean difference (95% CI)	
						Exposed versus internal references	Exposed versus external references
Birth weight	Exposed to EDC	582	3,553	3,531	3,479–3,583	–5.42 (–76,9 – 66,1)	35.0 (−17,2 – 87,2)
	Internal reference	620	3,500	3,537	3,487–3,586	.	.
	External reference	346,544	3,500	3,496	3,494–3,499	.	.
Birth weight at week 40	Exposed to EDC	117	3,580	3,566	3,462–3,671	–3.2 (−141 – 136)	−21.5 (−126,4 – 83,5)
	Internal reference	118	3,565	3,570	3,479–3,660	.	.
	External reference	76,277	3,550	3,588	3,584–3,592	.	.

In analyses adjusting for gestational age and potential confounders, women potentially exposed to EDC had children with a higher birth weight when compared to the external references (in average 63 g higher, 95% CI: 22–101), but not when compared to internal referents (in average 37 g higher, 95% CI: −19–93).

The proportion of preterm births among the women potentially exposed to EDC was marginally larger than the proportion of preterm births among the women in the internal reference group (Table 4), but the difference was not statistically significant. There were no difference in the proportion of preterm births between the women potentially exposed to EDC and the external references.

**Table 4:** Odds ratio with 95% confidence interval (CI) for preterm birth among women potentially exposed to endocrine disrupting chemicals (EDC) compared to the internal and external references respectively

	Preterm birth		Term birth					
	n	%	n	%	OR	95% CI	OR	95% CI
Exposed to EDC	46	7.9	536	92.1	1.53^a^	0.91–2.57	1.02^b^	0.73–1.43
Internal references	36	5.8	584	94.2	1	.	.	.
External references	27,815	8.0	318,729	92.0	.	.	1	.

^a^Exposed compared to internal references

^b^Exposed compared to external references

There was no difference in the proportion of children with low birth weight born to the women potentially exposed to EDC compared to the internal references (Table 5). Compared to the external references, the proportion of children with low birth weight was marginally lower among the women potentially exposed to EDC. This lower risk of low birth weight was likewise not statistically significant.

**Table 5:** Odds ratio with 95% confidence intervals (CI) for low birth weight among women potentially exposed to endocrine disrupting chemicals (EDC) compared to the internal and external references respectively

	Low birth weight		Normal birth weight					
	n	%	n	%	OR	95% CI	OR	95% CI
Exposed to EDC	21	3.6	561	96.4	0.98^a^	0.40–2.39	0.76^b^	0.41–1.37
Internal references	22	3.6	598	96.4	1	.	.	.
External references	16,330	4.7	330,214	95.3	.	.	1	.

^a^Exposed compared to internal references

^b^Exposed compared to external references

A sensitivity analysis only including first births did not show any differences of importance for gestational age and birth weight between the three exposure groups (data not shown).

## Discussion

In this prospective follow-up study of pregnant women referred to an OHC for counselling, we found no indications of reduced birth weight or increased risk of preterm birth among women potentially exposed to EDC. Women potentially exposed to EDC had children with a higher birth weight when compared to the external references. This might reflect an anabolic effect of EDC (for instance an estrogenic effect), but without consistent findings in analyses using the internal references, we consider this a chance or spurious result.

Women referred to an OHC are presumed to represent a high risk group, and constitute thereby a preferable basis for investigation compared to a population based study. The present study design enables comparison between women referred to an OHC potentially exposed to EDC and women referred to an OHC but unexposed to EDC. These two groups are highly comparable, since they are expected to be similar in most aspects except exposure status. Furthermore, use of register data provides a large study population contributing to enhanced statistical power.

These reassuring results can be understood in several ways. First of all, Denmark has a long tradition of worker protection regulation and the Danish Working Environment Authorities regularly performs inspections at Danish workplaces. Thus, potentially harmful chemical exposures of pregnant women at Danish workplaces are in general considered well controlled and chemically induced birth defects are rarely notified to the National Board of Industrial Injuries [20]. Therefore, the women defined as potentially exposed to EDC in the present study might not have been exposed to EDC to an extent that is strong enough to cause detectable adverse effects on gestational age and birth weight. A second explanation of the findings might be that the risk imposed by EDC is reduced by occupational risk assessment and counselling. If the risk assessment indicates a harmful exposure, actions to reduce or eliminate the exposure are recommended. Finally, awareness of potential harmful exposure, induced by the referral to an OHC, might cause a behavior change to a more protective lifestyle during pregnancy [13]. However, human embryo development is most vulnerable to toxic substances and endocrine disruption in the early embryonic period during the first 8 weeks of gestation, while counselling at an OHC usually takes place between 8 and 12 weeks of gestation.

One limitation of this study concerns the determination of exposure to EDC. The risk of EDC might go undetected in the present study due to misclassification associated with determination of exposure status. Using a JEM does not necessarily give an accurate picture of exposure since exposure might vary within job titles [7].

The JEM of van Tongeren et al. divides the chemical substances into seven substance categories [1]. In the present study all substances were combined into one group of EDC. By doing so, some accuracy in relation to potential differences between substances is lost. However, these reductions of categories were made to address the purpose of the present study - to investigate the general potential effects of EDC on gestational age and birth weight.

Finally and importantly, EDC are also found in many consumer products and the exposure besides the occupationally induced might be profound which would diminish the association in the present study. We know very little about what factors pregnant women are exposed to at home [21].

## Conclusion

Pregnant women with potential occupational exposure to EDC were not at increased risk of delivering preterm or low birth weight babies. Findings may reflect that exposure levels are low without impact on the studied reproductive outcomes or that occupational counseling result in effective preventive actions.

## Abbreviations

CI: Confidence intervals
CO80: Categories of occupation
EDC: Endocrine disrupting chemicals
ISCO: International standard classification of occupation
JEM: Job-exposure matrix
OHC: Occupational health clinic
OR: Odds ratio
PCB: Polychlorinated biphenyls
PIN: Personal identification number
SES: Socioeconomic status
